# Migratory Bone Marrow Edema Syndrome of the Hips: A Case Report

**DOI:** 10.5704/MOJ.1711.006

**Published:** 2017-11

**Authors:** A Santoso, PS Ingale, KS Park, TR Yoon

**Affiliations:** Department of Orthopaedic and Traumatology, Sebelas Maret University, Solo, Indonesia; ^*^Center for Joint Disease, Chonnam National University Hwasun Hospital, Gwangju, Republic of Korea

**Keywords:** bone marrow edema, hip joint, migratory

## Abstract

Migratory bone marrow edema syndrome (BMES) of the hip is a rare entity. We report the case of a 41-year old male with migratory BMES of the hip with eight months interval period between onset of the pain and consultation. This patient was successfully treated non-surgically. It is important to always inform the patient with unilateral BMES of the hip regarding the possibility of future involvement of the contralateral hip.

## Introduction

Bone marrow edema syndrome (BMES) of the hip is an uncommon disease which is characterized by a self-limiting process^1^. It primarily affects middle-aged men, and women during the last trimester of pregnancy^1,2^. BMES of the hip could present as a unilateral, simultaneous bilateral or a sequential/migratory case^3^. The migratory case is rare. We present a patient with a migratory BMES of the hips in association with a short review of previous similar case reports.

## Case Report

A 41-year old male came to our outpatient clinic with a one month history of right inguinal pain. The pain was localized, non-radiating, aggravated by activity and relieved by rest. Visual analog scale (VAS) was 7-8. There was no history of pain in any other joint, history of trauma, alcohol abuse, steroid medication or other related illness. He was a physically fit person and worked as an office staff. His body weight was 78kg and height 178cm with body mass index 24.6 kg/m^2^. On physical examination, there was a limping gait and positive on the Patrick’s test of the right hip. The right hip range of motion was: flexion 90°, extension 10°, abduction 20°, adduction 20°, internal rotation 20°, and external rotation 30°. Examination of the rest of his body including the spine and contralateral hip was normal.

**Fig. 1:**
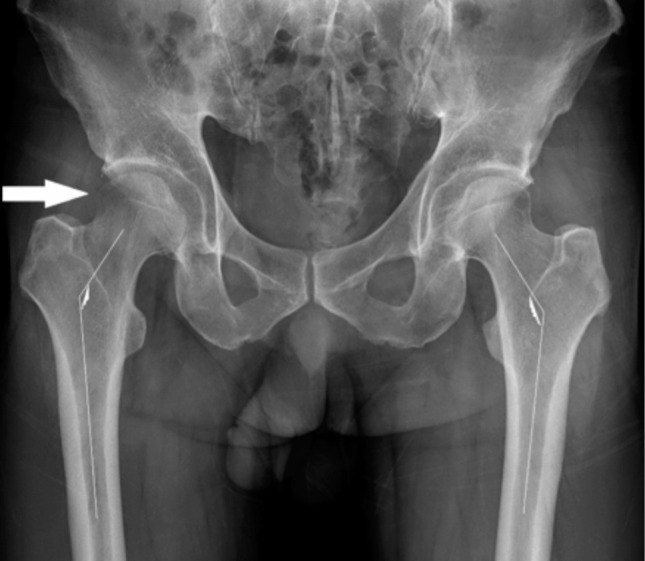
Anteroposterior pelvic radiograph showing osteopenic bone on right femoral head and neck (white arrow). Valgus neck-shaft angle indicated by the lines.

**Fig. 2:**
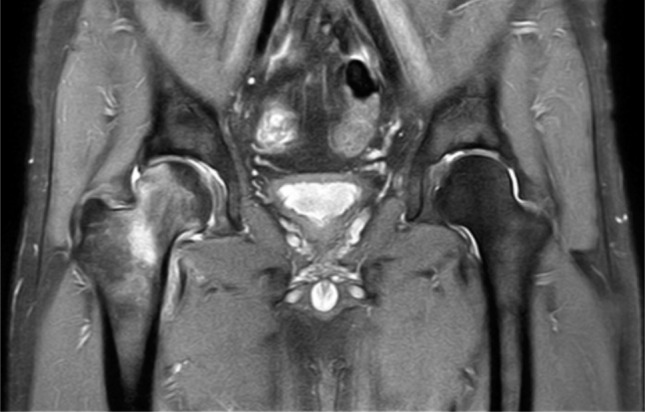
T2-weighted MRI showing high signal intensity on right femoral neck and head along with joint effusion.

Laboratory study including hematology, biochemistry, coagulation, erythrocyte sedimentation rate and C-reactive protein revealed normal values. A pelvic anteroposterior radiograph showed mild osteopenia on the right femoral head and neck with joint space preserved. He had bilateral coxa valga with neck-shaft angle on the right of 145° and left 145° (normal: 125-135°) (Fig. 1). T2-weighted magnetic resonance imaging (MRI) showed high signal intensity which involved the right femoral head and neck with joint effusion (Fig. 2). Bone mineral density (BMD) examination with dual energy radiograph absorptiometry (DEXA) showed the total bone density of the hip was 1.014 g/cm^2^ (T-score 0.6, Z-Score 0.6). Bone scintigraphy (Tc-99m) examination revealed increased uptake on the right hip centered on the femoral head. A diagnosis of BMES was arrived at based on clinical and radiological findings. He was managed symptomatically with an advice to rest, partial-weight bearing with crutch walking and analgesia for pain control (45mg of Pelubiprofen and a combination of 162, 5mg Acetaminophen +18,75mg Tramadol) twice daily. Monthly radiographic examination during follow-up was planned. Clinical improvement was noticed at one month and subsequently the patient felt relieved of all symptoms.

**Fig. 3:**
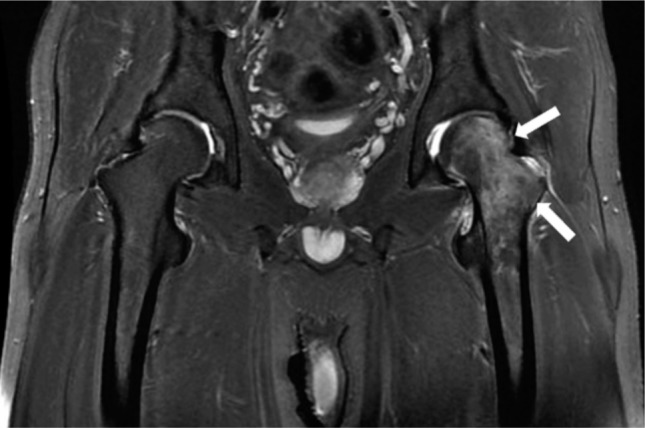
T2-weighted MRI at the second onset left hip (8 month after first onset in right hip) showing high signal intensity on left femoral neck and head (white arrows) with normal signal intensity on right side.

About eight months after the initial onset, the patient visited our outpatient clinic complaining of a new onset of pain (VAS 6-7) on the opposite hip. He complained symptoms similar to that on his contralateral (right) hip previously. There was no history of significant event before onset of the recent pain. On physical examination of the right hip, there was no pain and range of motion of the hip was full. Physical examination of the left hip revealed positive Patrick’s test with limitation of range of motion. As before, laboratory examination revealed normal results. Pelvic anteroposterior radiograph showed slight osteopenic area on the left femoral head and neck. T2-weighted MRI showed high signal intensity on left femoral head and neck with normal intensity on the right side (Fig. 3). The total bone density was 1.019 g/cm^2^ (T-score 0.6, Z-Score 0.6). At this second presentation, bone scintigraphy was not obtained. Due to similar clinical and image findings with previous contralateral right hip, we diagnosed this patient with having a migratory type of BMES of the left hip. Similar treatment protocol was instituted for the patient. Two months later pain had subsided and he had regained his normal activity level. He had no complaint on further follow-up at six months. Informed consent was obtained from the patient and his family for publication of this case report.

## Discussion

We report migratory bone marrow edema syndrome of the hips occurring in a middle-aged male patient with less than a year of interval of onset between the two hips. Previously, several migratory BMES of the hip cases have been reported^1-5^. Interestingly, all of them were of the male gender, with age range between 20 to 52 years. A wide range of the interval period between onset in both hips has been reported. The shortest interval was four months in a case reported by Garzon *et al*^1^, while the longest interval period was 12 years as recently reported in a case by Iannò *et al*^3^ (Table I).

**Table I:** Comparison with the previously reported cases of migratory BMES of the hip

Authors	Sex/Age (years)	Hip migration	Interval period	Possible risk factors	Treatment
Garzón G et al^1^	M/34	Right to left	4 months	Unknown	Conservative therapy (?)
Dhaliwal J et al^2^	M/20	Left to right	6 months	Hormonal therapy due to hypothyroidism	Analgesia
Iannò B et al^3^	M/44	Left to right	12 years	Repetitive trauma (left), Unknown (right)	Magnetotherapy + Calcitonin Inj. (left)/Sodium Clodronate Inj. (right) + non-weight bearing
Yi SR et al^4^	M/52	Right to left	3 years	Unknown	Analgesia + limited physical activity
Bolland MJ^5^	M/32	Right to left	6 months	Carbamazepin treatment due to epilepsy	Non-weight bearing
Present case	M/41	Right to left	8 months	Bilateral coxa valga (?)	Analgesia + partial-weight bearing

* M: Male, Inj: Injection

Several risk factors have been proposed to be associated with BMES of the hip including: repetitive trauma, heavy lifting occupation, steroid intake, alcoholism and hormonal/ neurovascular problem associated with pregnancy^2,3^. One of the five previously reported cases had a history of hypothyroid state and had received thyroxin supplement^2^, while the other had a history of long term carbamazepine treatment^5^. Our patient had bilateral coxa valga based on radiographic findings (neck-shaft angle >135° on both hips). Although a valgus hip is known to be associated with an increase in hip joint reaction force, its role as the risk factor for bone marrow edema syndrome remains unknown.

Symptomatic treatment remains the best choice for uncomplicated bilateral BMES of the hip. Treatment modalities from oral analgesic medication, oral/parenteral bisphosphonate, parenteral calcitonin to magnetotherapy have been reported^3,4^. The use of antiresorptive agent (bisphosphonate/calcitonin) is controversial, as osteoporosis was not always present in the BMES of the hip cases. Bone mineral density was within normal limit in our current case. Yi *et al*^4^ noted osteopenia of both hips on BMD measurement. Bolland^5^ reported an inconsistent finding with only one hip showing osteoporosis with the other one normal. Despite different result on BMD measurement, our present case and the case reported by Yi *et al*^4^ and Bolland^5^ were succesfully treated with only protected weight bearing or in combination with analgesic medication. The use of antiresorptive agent may be more beneficial for those who failed with symptomatic treatement and for those with a high risk of fracture^5^.

In summary, it is important to always warn the patient with a unilateral BMES of the hip on the possibility of future contralateral hip involvement. We hope this report, along with the short review of previously reported cases, may have some value in understanding the migratory pattern of BMES of the hip.
